# Staff Training in Autism: The One-Eyed Wo/Man…

**DOI:** 10.3390/ijerph13070716

**Published:** 2016-07-16

**Authors:** Karola Dillenburger, Lyn McKerr, Julie-Ann Jordan, Mickey Keenan

**Affiliations:** 1 Centre for Behaviour Analysis, Queen’s University Belfast, Belfast BT7 1NN, UK; l.mckerr@qub.ac.uk (L.M.); ja.jordan@qub.ac.uk (J.-A.J.); 2 School of Psychology, Ulster University at Coleraine, Londonderry BT52 1SA, UK; m.keenan@ulster.ac.uk

**Keywords:** Autism Spectrum Disorders, mental health, staff training, adults with autism, services, United Kingdom, Northern Ireland

## Abstract

Having well-trained staff is key to ensuring good quality autism services, especially since people affected with autism generally tend to have higher support needs than other populations in terms of daily living, as well as their mental and physical health. Poorly-trained staff can have detrimental effects on service provision and staff morale and can lead to staff burn-out, as well as increased service user anxiety and stress. This paper reports on a survey with health, social care, and education staff who work within the statutory autism services sector in the UK that explored their knowledge and training with regards to autism. Interview data obtained from staff and service users offer qualitative illustrations of survey findings. Overall, the findings expose an acute lack of autism-specific training that has detrimental impacts. At best, this training was based on brief and very basic awareness raising rather than on in-depth understanding of issues related to autism or skills for evidence-based practice. Service users were concerned with the effects that the lack of staff training had on the services they received. The paper concludes with a discussion of policy routes to achieving quality staff training based on international best practice. The focus is on improving the quality of life and mental health for services users and staff, as well as making potentially significant cost-savings for governments.

Data reported here were collected as part of a larger project entitled “Helping the most vulnerable out of the poverty trap and reducing inequality: Policies, strategies, and services for individuals with Autism Spectrum Disorder, including intellectual and neuro-developmental disabilities”, that was funded by the Office of the First and Deputy First Minister (OFMDFM) in Northern Ireland. Full reports are available elsewhere [1]. The views expressed are those of the authors.

## 1. Introduction

The number of people diagnosed with autism is on the rise. The estimated prevalence rate of autism across all age groups is now approximately 1 in every 68; for children, prevalence is even higher, with at least 2% of all children are affected in the USA [2]. However, recent data indicate that the prevalence rate may be even higher. For example, prevalence rates in South Korea were reported to be 2.64% [3] and in Northern Ireland, 2.3% of school children (6–15 years of age) have been diagnosed with Autism Spectrum Disorder (ASD) [4]. In the United Kingdom (UK) as a whole, the Millennium Cohort Study (*n* = 13,287 families) revealed that 3.5% of 11-year-old children are reported to have autism, i.e., parents had been told by professionals that their child has autism [5]. These high prevalence figures are supported by self-reports of 16-year-old young people (*n* = 1034) in Northern Ireland of whom 3.1% stated that they had autism and by 11-year-olds (*n* = 2319) of whom 2.7% reported to have autism [6]. There are no published prevalence rates for adults with autism, although prevalence rates are likely to be similar to those for children [7].

ASD is diagnosed on the basis of pervasive difficulties in communication and social interaction, as well as restricted interests and inflexibility in behaviours, that are observed from an early age in most cases [8]. The term ‘autism spectrum’ refers to the fact that the level of ability and function, i.e., the impact and experience, varies significantly between individuals. Approximately 50%–75% of individuals with ASD have co-occurring intellectual disabilities and/or other neurodevelopmental disabilities, and/or mental health problems, such as hyperactivity, depression, or anxiety [9,10].

Without appropriate interventions, children with autism will become adults with autism with limited academic, social, and life skills [11,12]. A 40-year follow-up of adults (*n* = 65; mean age 44 years) diagnosed with autism as children in the 1970s indicated that 75% had unchanged IQ levels (≥70) since their childhood assessment and only minor improvements in terms of language, while 25% of them could not be assessed due to challenging behaviours; they had not developed language above their level as a three-year old [13]. The authors concluded that “[a]lthough many attended specialist autism schools as children none had access to the intensive, early behavioural programmes that are available today” (p. 56; cf., [14]). Ultimately, the use of pharmacological restraints may seem the only intervention left [15].

Physical health problems, such as vision impairment, epilepsy, gastrointestinal problems, and food allergy are common, as are sleep disorders, communication problems, and challenging behaviours [16,17]. Adults with autism are more likely to die prematurely compared with the general population or familial controls without autism [18]. Despite the difficulties listed above, some people argue that autism is a neurodiversity that should be celebrated [19] and that interventions necessarily involve some sort of normalising agenda; incidentally, this is viewed with negative connotations [20,21].

Parents of children with autism tend to experience more mental health issues than parents of other children [22,23,24,25]. Providing lifespan care for adult sons/daughters with autism places a huge burden on aging caregivers [26,27] that can impact negatively on the mental health of families [28]. In addition, given that the “cost of autism” for governments is higher than the cost of cancer, strokes, and heart disease combined, i.e., annually £34 billion in the UK [29], parenting a child with autism throughout the lifespan can lead to economic exclusion [26,27,30].

Not surprisingly, most individuals with autism and their families require specific autism services as well as the full range of other health, social care, and educational supports. In Northern Ireland, the lack of support for people with autism has led to severe mental health difficulties (34%), depression (57%), and anxiety (65%) in adults with autism [31]. School census data reveal that children with autism have poorer educational attainment rates than typically-developing peers [32] and they are bullied more frequently [33]. Multiple deprivation measures show evidence that families affected by autism experience higher expenses and lower economic incomes than other families [34].

Consequently, individuals with disabilities, including autism, require not only disability services but also the full range of other health, social care, and educational supports. There is some evidence that staff in these services are ill-prepared to support individuals with autism [35,36,37], however, the extent of unmet staff training needs remains unclear [38].

Courses that lead to initial professional qualifications in general offer very little by way of training in autism. For example, during their initial training speech and language therapists (SLT), occupational therapists (OT), psychologists, nurses, and other allied health and medical professionals will have received a maximum of 1–3 h of lectures (depending on where they have studied), while most other qualifying courses for social workers and teachers offer no training in autism or appropriate interventions at all [39].

This paper focuses on training for professionals after they have qualified (i.e., post-qualifying training). We report findings regarding knowledge, training, and practical experiences of health, social care, and educational professionals across a variety of autism related settings. While most previous research explored training methods or professional views [40,41], we surveyed professionals for a wide variety of relevant professions about their level of autism related staff training, and interviewed service users and staff about their ensuing experiences; that is, we asked service users, including adults with autism and parents of children with autism, about their views on staff training, service delivery, and inclusive practices. Thus, this paper elucidates the lived experience of those who use, and those who are tasked with providing, autism services.

## 2. Methodology

### 2.1. Participants

#### 2.1.1. Survey Participants

A total of *n* = 798 professionals from health, social care, and education took part in an online survey (more details under research instruments). The majority of participants were health and social care staff (71%; *n* = 569), with smaller numbers of general education staff (10%; *n* = 78, of which over half (*n* = 43) were school teachers), and Further and Higher Education staff (8%; *n* = 65). The age range of professional participants was 18–65 years of age. The average age for the different professional groups was 40–46 years of age. 

**Figure 1:**
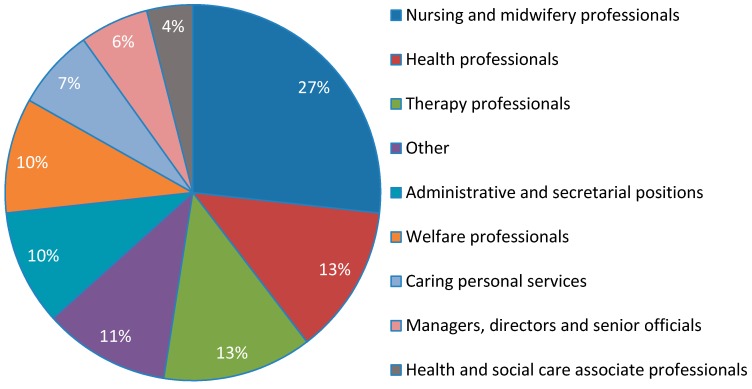
Occupations of health and social care respondents.

Figure 1 shows the occupational breakdown of health and social care participants. Almost one third of the participants were nursing and midwifery professionals (27%), while others included health professionals, e.g., medicine and psychology (13%), allied health therapy professionals, e.g., occupational therapy, speech and language therapy and physiotherapy (13%), welfare or social services professionals, e.g., social work (10%), and administrative and secretarial staff (10%), and others (11%).

**Figure 2:**
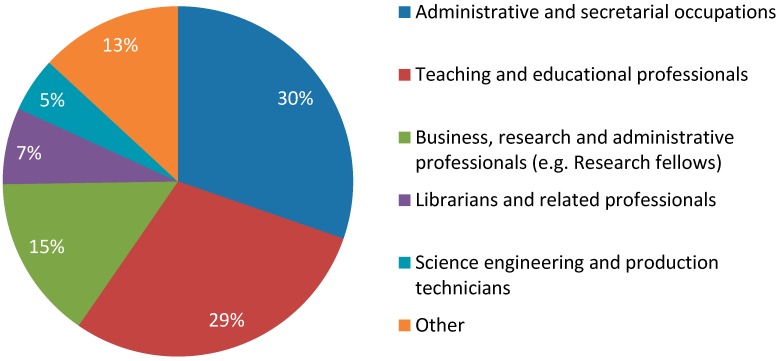
Occupations of Further Education and Higher Education participants.

Figure 2 shows the occupational breakdown of Further Education and Higher Education (FE/HE) staff. The majority of these participants (89%) were from Higher Education and, overall, most respondents were employed either in administrative/secretarial (30%) or teaching and educational (29%) professions.

#### 2.1.2. Interview Participants

Individual interview participants included the following three groups:

- Five adults with autism (three men and two women) aged 18+ years of age;
- 14 parents/caregivers (including two couples); the son(s)/daughter(s) of five of these parents were teenagers or young adults with autism; the son(s)/daughter(s) of the other nine parents were younger;
- 12 education professionals; three of the education professionals were parents of son(s)/daughter(s) with autism, while nine of the education professionals did not have a child of their own with autism.

### 2.2. Research Instruments

In the UK, post-qualifying staff training in autism is categorised in along three distinct levels or tiers [42];

- Level /Tier 1 equates to brief autism awareness sessions, usually lasting no more than 1–2 h aimed at general autism service providers and other frontline staff.
- Level/Tier 2 usually takes the form of a half-day seminar aimed at staff who work directly with a child or adult with autism.
- Level/Tier 3 commonly takes 1–2 days and is aimed at building on existing knowledge for staff who are taking a lead in autism service provision.

Health and social care trainers tend to use the term ‘Level’, while some others use the term ‘Tier’ to differentiate these levels of basic autism training. Some departments may not use the same terminology, however, their training intensity levels concur roughly with the NAS levels/tiers. Throughout this paper, the term ‘Level’ will be used. Generally, this training is provided by employers ‘in-house’ or contracted from the voluntary sector. For more advanced training, universities offer Masters and Doctoral level training in autism as well as Behaviour Analyst Certification Board-approved Masters level course sequences [43].

#### 2.2.1. Professional Online Surveys

The online surveys were based on the Professional Autism Needs Questionnaires (PAN-Q) [28,44]. The survey questions focused on information about autism training, professional practice, and respondents’ recommendations for the future direction of services for individuals with autism. The survey enquired about the three training levels/tiers and about any other training received (for full questionnaires see [1]; available free online).

The surveys were the same for health, social care, and education sectors, but the wording of some of the questions was adjusted slightly to reflect the different target professions (e.g., the term ‘teacher’ was replaced with the term ‘medical professional’). All surveys were previewed by the services user/professional steering group and pilot tested; no substantial changes were necessary. Survey questions were available in alternative formats (e.g., hard copies) on request. No such requests were received.

#### 2.2.2. Semi-Structured Interviews

Interview questions were modelled on Parent Autism Needs Questionnaire (FAN-Q) [28,44]. Questions were open and semi-structured, thus allowing for supplementary questions, where needed. Prior to finalising the interview format, the questions were reviewed by a steering group comprising adults with autism, parents of children with autism, and autism professionals from the voluntary sector. Pilot testing revealed that no changes were necessary (for full list of questions see [1]; available free online). Interviews were carried out by two of the authors (Lyn McKerr and Julie-Ann Jordan), who were highly experienced interviewers. Both had police clearance (i.e., AccessNI) and had undertaken training regarding conducting qualitative interviews, focus groups, and held certificates in Good Clinical Practice.

### 2.3. Research Procedures

#### 2.3.1. Professional Online Survey

The surveys were hosted on SurveyMonkey^®^ (SurveyMonkey, Palo Alto, CA, USA). Links to the surveys were distributed by email to gatekeepers, who were asked to circulate the link directly to their staff/members via emails, staff newsletters, or online using staff intranet services. Hardcopies were available on request.

For Health and Social Care staff, the link was sent to the five regional Health and Social Care Trusts (HSCT), as well as the Department of Health and Social Services and Public Safety (DHSSPS) in Northern Ireland for onward circulation to staff. Most of these staff work in children’s services. The whole-time equivalent (WTE) of staff who work with adult clients with autism is extremely low; at the time of the survey there were only seven WTE professionals dedicated to providing adult autism diagnosis and services in a country with a total population of over 1.8 million people [45,46]. 

Given that the survey predated the establishment of the new NI Education Authority (a restructuring of Education and Library Boards), links to the survey for education staff were sent to all Education and Library Boards (ELB) and the Department of Education (DE) and professional organisations for onward circulation to staff. Links to the online survey for voluntary sector staff were distributed to all the main autism charities in Northern Ireland for onward circulation. 

#### 2.3.2. Semi-Structured Interviews

Invitations to participate in semi-structured interviews were sent to autism voluntary groups and distributed via social media. Potential participants were invited to contact the researchers by telephone or email and those who volunteered were provided with background information on the project and contact details before being contacted to arrange a suitable time, location, and date for interview. 

Individual interviews were conducted either in the participants’ home or office, whichever was preferred. Each participant was required to read the information sheet and sign a consent form prior to commencing the interview. Only participants who gave signed consent took part. All but three interviews were audio-recorded. Where consent for audio recording was not given, the interviewers made contemporaneous notes in lieu of recording. All audio recordings were subsequently transcribed and anonymised.

### 2.4. Data Analysis

Survey data were analysed using SPSS software [47]. Interview data were analysed using thematic analysis [48,49]; two of the authors (Lyn McKerr and Julie-Ann Jordan) independently coded the qualitative data obtained in the interviews and subsequently confirmed codes with each other for consistency.

### 2.5. Ethics Statement

All subjects gave their informed consent for inclusion before they participated in the study. The study was conducted in accordance with the Declaration of Helsinki, and the protocol was approved by the Ethics Committee of School of Education, Queen’s University Belfast 12/09/2012.

## 3. Results

### 3.1. Professional Autism Knowledge and Training

**Figure 3:**
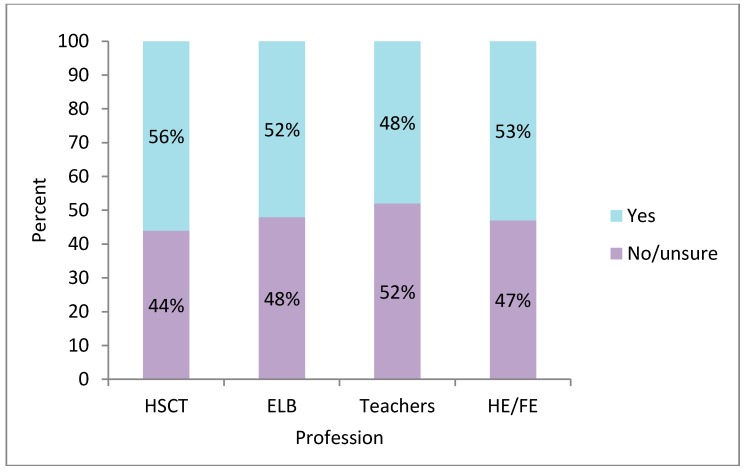
Personal knowledge of an individual with autism.

Over half (56%) of health and social care (HSC) respondents indicated that they knew someone with autism personally (i.e., a family member, friend, acquaintance, or colleague). The figure was similar for education professionals, i.e., Education and Library Board (ELB) staff, teachers and Further and Higher Education (FE/HE) staff (Figure 3).

The importance of personal contact was confirmed in the interviews, as parents of children with autism and adults with autism stated that they relied heavily on family, peers, or the voluntary sector for support:

*‘My mum and dad would have been good [for support], but my mum has recently been diagnosed with Alzheimer’s, and my dad, he goes into hospital every second week for treatment…so really, we don’t leave the kids with them at all now. And my sister, she’s got fibromyalgia. Her younger son, he’s got Asperger’s and the younger daughter, she’s not diagnosed but we’re pretty certain she’s got autism as well. She’s got enough going on in her house [without helping us].’* 

(Adult A’s wife)

Intra-family recurrence rates of autism are high (estimated to be up to 50%; [50,51], although the genetic links have not been scientifically established [52]. Of course, familial co-occurrence does not necessarily have to be negative, e.g., Adult A considered his own condition gave him a unique and beneficial insight into parenting a child with autism, and helped him support his partner when their son’s behaviour was causing problems:

*‘Yes, at times I’ve had to tell [wife] to just leave him alone. I’m like ‘Just leave him’ …and you just wait until he calms down, and tell him what he’s done wrong, he’s got to fix whatever he’s done…but at the same time, I probably don’t let him away with as much as [wife] will.’* 

(Adult A)

**Figure 4:**
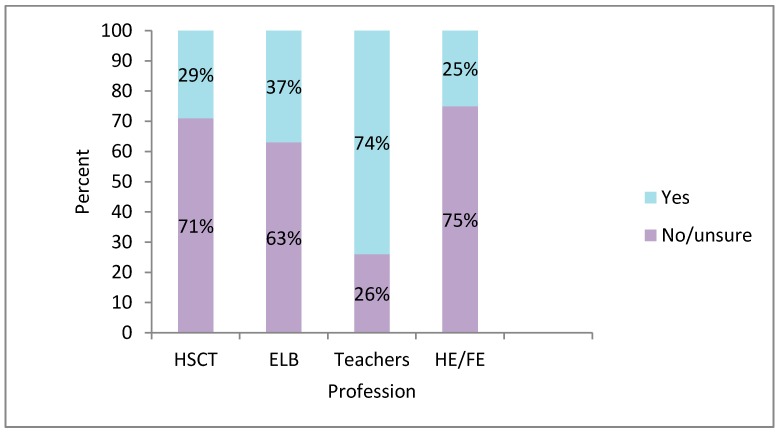
Percentage of professionals’ basic post-qualifying, in-service autism training.

Even so, all professional respondents had some contact with persons with autism and one fifth (21.8% of *n* = 40 ELB staff and 16% of *n* = 501 HSC staff) saw individuals with autism almost every day, autism training was optional. Figure 4 shows that fewer than one-third (29%) of health and social care (HSC) staff had received basic post-qualifying, in-service autism awareness training (i.e., Level 1); the percentage was similar for general education (ELB) professionals. 

Although the response rate for teachers was relatively small (*n* = 43), all of them taught children with autism at some point, while two thirds of them (61.9%) saw students with autism almost daily. Yet, only three quarters (74%) of the teachers stated that they had received some kind of autism training. As for Further Education/Higher Education (FE/HE) professionals, 7% saw students with autism almost daily and 17% engaged with students with autism at least once a month; a quarter of FE/HE professionals had received some autism training. None of the participants had received autism training before they started working with individuals with autism. 

A substantial number of interviewees thought that autism training should be mandatory for all staff who are working with individuals with autism, in particular in the health and social care sector, where people with autism access a wide range of core services, such as doctors and hospitals, social services, as well as mental health and other therapy services. Suggestions also were made that autism training should be provided early in the careers of professionals, offered regularly to all staff, and be easy to access:

*‘I feel training and updates should be mandatory as we encounter people with autism frequently as service users and some colleagues.’* 

(HSC participant)

Some respondents thought that it would be useful to have a designated ‘expert’ who they could consult for advice. It was also felt that there should be service-user involvement in training, where possible. Most participants felt that autism training should be either incorporated into professional (pre-qualifying) training or form part of staff induction:

*‘I think it should be included in basic nursing training. We are not always made aware when referrals are put through that there has been a diagnosis of autism and training would assist us to recognise it.’* 

(HSC professional)

Table 1 shows the proportion of respondents who had received autism training; this is further broken down into proportions of total respondents who had undertaken specific training at Levels 1, 2, and 3. These data indicate that, even where staff had undertaken some autism training, the training levels were low, i.e., for 19% of health and social care (HSC) staff, 27% of general education staff (ELB), 12% of further and higher education (FE/HE) staff, and nearly half of the teachers (47%) post-qualifying autism training was limited to Level 1 (i.e., 1–2 h autism awareness raising). Only 10% of HCS respondents, 28% of ELB staff, 13% of teachers, and 5% of FE/HE staff had undertaken Level 2 autism training (i.e., one half-day seminar). Only 3% of HSC staff and 19% of general ELB staff had attended Level 3 training (i.e., 1–2 day seminars). None of the participants had undertaking post-qualifying autism training at Masters or Doctoral levels.

When asked about future needs, staff recognized that training in adult autism services was even sparser than in children’s services, in particular with regards to mental health services:

*‘Better training for staff working in mental health services on working with people with autism. This is important as Asperger’s Syndrome now falls within mental health services, whereas previously this came under learning disability services.’* 

(HSC professional)

A total of *n* = 175 health and social care (HCS) professionals responded to an open question about their future autism training needs, with only a very small minority (*n* = 12) having no suggestions or being happy with the training that had received. The vast majority (*n* = 163) felt that more training was needed. Proposals for improvements in staff training focused on the organisation (i.e., when and how training was delivered) as well as the content of this training.

### 3.2. Service User Views about Staff Training

The shortcomings of existing staff training became even more apparent in service users’ interviews, which identified key themes of diagnostic delays, insufficient professional knowledge, and statutory support. Perhaps unsurprisingly, given the concerns identified previously, parents, in particular, expressed worries about the future when they would no longer be able to ‘fill the gaps’ in the provision of care for sons and daughters with autism. The interviewees recognized that professionals were not trained adequately and did not have enough knowledge about autism. Adults with autism stated that they usually realised relatively early in their childhood that they had difficulties particularly in social situations and at school, but that these difficulties had never been addressed appropriately. Prior to receiving the diagnosis of autism, all of the adults who participated in the interviews had received adult mental health services related to issues that included anxiety and depression, self-harm, and/or alcohol abuse. 

Three of the adults with autism reported that they had young children with autism, and two had close relatives with autism, although not necessarily formally diagnosed. In fact, most of the adults with autism had sought their own diagnosis subsequent to their own child’s diagnosis:

*‘I went and saw the GP [General Practitioner], and I said to the GP ‘I believe I’m autistic, because of [son]’s diagnosis, that I match up quite a bit to how he is, and stuff.’* 

(Adult A)

Obtaining an assessment and diagnosis later in life was an important issue for these adults, however, there were extensive waiting times, often exceeding two years. All of the adult participants had thought that they were on the autism spectrum prior to seeking diagnosis. They all had experienced considerable difficulties throughout their life and complained about the lack of professional training in adult diagnostic techniques and absence of dedicated adult autism service:

*’…so the GP was like ‘we’ll try and get you someone to see in the Psychology department’ and he says ‘but I daren’t put autism down because they might just simply say “Oh, we don’t diagnose for that”, and you wouldn’t get seen for any trouble’, so it took a long time for that…* 

(Adult A)

For two of the women with autism it was even more difficult to obtain a diagnosis than for the men and they felt that diagnosticians had a very ‘stereotypical’ male-oriented view of autism:

*‘The GP wrote off…but suggested that because I was married and had a job there was ‘No hope [of a diagnosis]’…I took an ADOS8 test, and an IQ test. They said ‘no’…I took the whole day off work to attend [HSC clinic]. [HSC diagnostician] said, if I had Asperger’s I wouldn’t have done that, but I was so anxious…’* 

(Adult D)

The lack of professional knowledge about how the behaviour of girls with autism differs from that of boys led to rather clichéd views of the condition. Consequently, female adult participants sought and paid for autism diagnosis outside the system, through private providers. This was also true for the girls whose parents took part in interviews. 

Parents reported that they had been aware of problems with their daughters, but felt professionals did not have the experience or appropriate knowledge and training to diagnose autism in girls. One of the parents reported that they had paid for private assessments for two daughters to avoid the stress of diagnosis from the statutory sector. The older daughter was diagnosed after leaving school, but the younger child’s private diagnosis was not accepted in the statutory education sector and consequently support was not provided. The younger child had significant difficulties in school and withdrew socially:

*‘The autism service in [education authority] wouldn’t talk to the parent or the child, only to the school. Teachers at school just don’t understand…Girls have a different profile, so you have autism, and Asperger’s, and Asperger girls etc…The multidisciplinary teamwere failing to protect my child.’* 

(Parent of teenage daughter with autism)

At times parents who had paid for a private diagnosis also sought confirmation from statutory clinicians, thus exposing the child to the diagnosis process repeatedly. Usually, the private diagnosis was confirmed:

*‘Yes, we suspected from about 15 to 18 months that [name] had autism. It took us quite a while to get her on to the waiting list to be diagnosed, so we actually took her privately ourselves…[t]hen we got her on to the waiting list to be diagnosed’* 

(Parent of preschool daughter with autism)

Not all adults who sought and received an autism diagnosis requested or required access services:

*‘Initially I didn’t sort of go seeking diagnosis immediately…it probably wouldn’t be from the point of view of seeking further services or intervention.’* 

(Adult B)

Overall, adults with autism and parents of children with autism felt that professionals were poorly trained or ill-informed and as a consequence were not able to support their children. The same was true for adults with autism, particularly when the person with autism was cognitively competent, articulate, and able to function in life. Only one participant was entirely satisfied with the statutory support he had received:

*‘Yes, I still have my therapist…she has been with me for maybe 2 or 3 years…and she can keep an eye, she can support me.’* 

(Adult C)

Most participants were not satisfied, pointing out that necessary staff training or skills were not in place and there was limited access to required services:

*‘I think there probably is a significant unmet demand, including among people who have a much greater need for services or support than I would.’* 

(Adult B)

Parents agreed that there should be more specialist training for general health service providers, such as doctors and dentists [53,54]. Without staff autism training, one parent explained how, instead of receiving free dental care for their child, the family incurred substantial additional expenses:

*‘…there’s a thing they should do, get autism dentists!…Our dentist said ‘I cannot deal with that’ and referred us to this private dentist, and we thought it would be a couple of hundred pounds…£600 I think he charged us, for fillings and X-rays, he gave him like a whole work-up once he got us in there.’* 

(Parent of two young adults with autism)

While not every person with autism requires social care services, those who do were very dissatisfied with the availability and quality of services.

*‘I’ve heard tell there are such things as disability social workers, I think they’re as rare as hen’s teeth, I think that would probably be of benefit.’* 

(Adult A’s wife)

*‘…there’s a cut-off age, she would no longer be his social worker, once he reached 16, but I don’t remember seeing [name] the last year, it might even have been [since he was] 14, or 12…because when he got to a certain age, she no longer was his social worker, because he didn’t fit the criteria.’* 

(Parent of adult daughter with autism)

*‘The contrast between what happens pre-19 and post-19 is a total disgrace, total disgrace… Shocking- the money just stops, and everything stops and [son] had severe mental health problems from when he knew he had to leave school…and then everything else dried up, all the money for his social life, everything.’* 

(Parent of adult son with autism and severe learning disabilities)

## 4. Discussion

Well-trained staff are crucial for those directly affected by autism who need support and intervention, as well as for their caregivers. A survey was carried out amongst health, social care, and educational professionals who work with individuals on the autism spectrum in the UK (specifically in Northern Ireland) to establish their training levels in relation to autism. In addition, in-depth interviews were carried out with some of these professionals and also with services users, namely, adults on the autism spectrum and parents of children with autism, to allow for triangulation of the survey findings and include the voice of services users with autism. 

Findings showed a severe lack of adequate training of professionals, i.e., most of the professionals having attended none or only minimal training session (i.e., 1–2 h). Critically, more than two-thirds of the professionals who took part in the study had not received any autism training at all and were aware of the subsequent stress and potential of burn-out caused by being asked to do a job that they were ill-prepared to do (i.e., high demands and low control; [55]).

Only one in ten of the professional participants had received Level 2 autism training (lasting ½ day). In fact, the term ‘training’ is misused in this context. Clearly, a short evening or lunchtime talk (i.e., Level 1) or even a half-day course (i.e., Level 2) should not be considered training, it merely serves as familiarization of the audience with some of the issues presented [56]. Put another way, “Would you trust a doctor to perform a C-section after he/she had listened to a couple of hours of a PowerPoint presentation?” [57]. The proper definition of ‘staff training’ should be defined as enduring change in behaviour [58] that is based on prolonged, in-depth study and the assessment of specific advanced skills [37,59].

This lack of proper training is a matter of great concern as health professionals are best placed to identify early signs of autism when young children present with developmental delays or communication disorders. This early ‘window of opportunity’ is important for putting targeted interventions in place to address and prevent many of the challenges that can come with autism [60]. In addition, while the prevalence of autism in school children has been rising by 0.2% per year in NI and presently is reported at 2.3% [4], obviously children and adults with autism use all core health services, such as accident and emergency services, mental health provision, oncology, surgery, and general medicine. A well-trained workforce can identify and minimise many of the issues that may cause undue stress, such as lengthy waiting times or noisy treatment areas [61]. 

Many of the professionals felt that with better training they would be better equipped to meet the needs of individuals with autism, yet they were neither cognisant of local universities’ advanced training in autism nor of advanced training in behaviour analysis [62]. It is important to note here that, internationally, behaviour analysis-based interventions are recognized as the gold standard in autism practice [63,64,65], although the science of behaviour analysis itself is not autism specific [66]. The various training levels in this science, that are approved by the Behaviour Analyst Certification Board [43], are increasingly recognized in the UK [67]. Research reported here confirmed earlier findings [68], that professionals were not as au fait with these issues as were parents [68]. 

Overall, professionals felt that training should be more accessible, delivered in a variety of formats, tailored to their specific roles, should contain more ‘real-life’ examples, and be informed by service users. While there is a sizeable amount of governmental rhetoric that staff training needs are being met [35,69], results reported here indicate that this clearly is not the case [70]. 

The survey showed that nearly half of the professional participants knew someone with autism personally. This makes a farce of brief 1–2 h autism awareness-raising sessions directed towards professionals who work in the autism field. They required and asked for much more advanced autism training [59]. Intriguingly, the percentage of professionals who knew someone with autism personally was somewhat lower than in the general population of adults (44%–52% vs. 51% for the general population; [71]. It was noticeably lower than the percentage of 16-year-old young people who knew someone with autism (72%) and only slightly higher than 11-year old school children who knew someone with autism (43%; [72]). It is disquieting that the general public (especially young people) may be more in touch with autism than professionals in autism services. 

Not surprisingly, service user interviews identified the lack of adequate staff training, inadequate services, and lack of support for individuals with autism and their caregivers and saw that this potentially contributed adversely to their mental health issues. Service user interviews also pointed towards a dearth of staff knowledge with regards to diagnosis, especially for women with autism. They confirmed that timely diagnosis was hampered by shortage of staff trained in autism diagnostic procedures [73,74].

Misguided attitudes about diagnostic instability of early diagnosis [75,76,77] have led locally to a ‘watch-and-wait’ approach [5] and adult diagnosticians were in extremely short supply in NI (i.e., one FTE per quarter of a million head of population). In addition, behaviour analytic programmes for early intensive intervention, challenging behaviours [78], and other mental health issues were not available in a statutory sector that relied heavily on the sparse provision of behaviour analysts in the voluntary sector (i.e., one FTE in the voluntary sector per half a million head of population) [79,80,81,82,83].

Subsequent to diagnosis, professionals were ill-prepared to respond to the complexity of needs of individuals with autism and their families, especially where multiple intra-family occurrences of autism and mental health issues were concerned. Given that existing mental health services were unable to provide for the growing number of services users with autism, the mental health of individuals on the autism spectrum is at higher risk than that of those not on the autism spectrum [53,84].

The interviews with parents showed that, due to lack of autism training of medical professionals, a number of them opted for private healthcare, putting additional burden on families already struggling, with the extra expenses further restricting other activities and opportunities for social inclusion [85]. Thus, individuals with autism experience a ‘two-tier’ system, where statutory providers do not meet their needs and families cannot afford to opt for necessary specialist private treatment. Where social and medical needs are not addressed adequately, health inequalities, social exclusion, mental health issues, deprivation, and challenging behaviours are likely to increase [78].

Many of these issues can be addressed through appropriate staff training [86] and, consequently, improved services [87]. For example, recent research on long-term outcomes has confirmed a statistically significant relationship between early intensive applied behaviour analysis-based interventions and optimal outcomes for autism, including a significant increase in adaptive skills and a reduction in autism symptoms, such as restricted and repetitive behaviours [88,89]. Therefore, they have the potential to mitigate against the deterioration in mental health [80] and consequently, improve employment prospects and lessen adult care needs [89,90] reducing costs to the economy, e.g., the reduction of lost employment would represent a 36% saving in the annual cost of autism for adults [29]. 

As mentioned earlier, these international findings stand in contrast to very poor long-term outcomes for adults with autism in the UK [13,14], where lack of recognition of behaviour analysis-based interventions is rife [91]. Having said this, the importance of ‘early intervention’ for individuals with autism has been recognised in the UK [92]. However, the definition of ‘early intervention’ is not coherent. In autism services in NI, for example, early intervention means a 1–3 h once-off visit shortly after the diagnosis, in which parents receive autism awareness-raising information and leaflets or are offered a few workshops [1], and, at best, a referral to a speech and language therapist for a very limited number of visits, while international best practice defines ‘early intervention in autism’ as 25–40 h per week, intensive, individually-tailored, behaviour analysis-based interventions that last at least two years or as long as deemed necessary [93,94].

Ultimately, given the very low uptake of staff training it is likely that the development of policies and practices across a wide range of services will be determined by staff and policy-makers without much training or appropriate experience with autism, thus, perpetuating problems identified some time ago, such as the ethically-dubious and controversial practice of people writing reports that include topics that are clearly outside their area of expertise [11,95].

As in any study, there were certain limitations in the research reported here. First, the dissemination of invitations to professionals to participate largely relied on gatekeepers, some of whom were able to reach more participants than others. It remains unclear if this was due to the information not being disseminated to potential participants by some gatekeepers or if the information reached potential participants who then decided not to take part. Furthermore, the research did not include an assessment of staff knowledge and competence prior or after training. Clearly, ‘training’ is only useful if it leads to competence in the relevant areas. Simply attending a talk is not the same as being trained. Training ‘evaluations’ commonly are conducted post hoc, immediately after the event and usually focus on enjoyment and organization of the event and friendliness of the staff, who delivered the event, they seldom assess the practical implementation of the newly learned content. Future research should focus on knowledge and skills acquisition [35].

## 5. Conclusions 

In sum, health, social care, and educational professionals are best placed to identify early signs of autism and to use this valuable ‘window of opportunity’ to put in place targeted and effective interventions. Lack of training leads not only to staff stress and burn-out [96,97], but more importantly, it increases services users’ and caregiver burden and risk of economic hardship and mental health problems [22,27,98]. Currently, staff training in the UK (as exemplified in NI) falls considerably short of requirements and does not meet international standards of best practice. Findings reported here show that in a culture where a lack of apposite training is the norm, those who attend minimal ‘autism training’ are regarded as ‘autism experts’ in the eyes of others who know even less. Dangerously, they may even become the trainers for the next generation. In reality, they are the one-eyed wo/man in ‘the land of the blind’ [99].

## Figures and Tables

**Table 1:** Proportion of professionals who completed autism training at Levels 1, 2, and 3.

Professional	Total Who Received Training ^1^	Level 1	Level 2	Level 3
Teachers	74%	47%	13%	0%
ELB staff	37%	27%	28%	19%
Private sector staff ^2^	30%	-	-	-
HSCT staff	29%	19%	10%	3%
HE/FE staff ^2^	25%	12%	5%	-

^1^ Proportions are calculated with ‘prefer not to say’/missing responses excluded; ^2^ Some cells suppressed due to small values for personal information—denoted by a dash.
